# Hidradenitis Suppurativa: Higher Tobacco Pack-Years in Patients with Metabolic Comorbidities

**DOI:** 10.3390/life15111647

**Published:** 2025-10-22

**Authors:** Yannik Haven, Nessr Abu Rached, Charlotte Michel, Daniel Myszkowski, Lennart Ocker, Ioannis A. Zeglis, Eggert Stockfleth, Falk G. Bechara

**Affiliations:** International Centre for Hidradenitis Suppurativa/Acne Inversa (ICH), Department of Dermatology, Venereology and Allergology, Ruhr-University, 44791 Bochum, Germany; charlotte.michel@rub.de (C.M.); daniel.myszkowski@kklbo.de (D.M.); lennart.ocker@kklbo.de (L.O.); ioannisalexandros.zeglis@klinikum-bochum.de (I.A.Z.); eggert.stockfleth@kklbo.de (E.S.); falk.bechara@kklbo.de (F.G.B.)

**Keywords:** hidradenitis suppurativa, smoking, tobacco pack-years, metabolic syndrome, comorbidities, HS

## Abstract

**Background**: Hidradenitis suppurativa (HS) is a chronic, relapsing inflammatory dermatosis with substantial quality-of-life impact. HS frequently co-exists with obesity and metabolic comorbidities. Cigarette smoking is highly prevalent and has been linked to heightened inflammatory activity and impaired wound healing. The role of cumulative tobacco exposure (packyears) in relation to metabolic comorbidities in HS is less well defined. We therefore investigated whether lifetime pack-years relate to laboratory parameters and the presence of comorbidities in HS. **Methods**: We conducted a retrospective, single-center study involving 131 patients with HS. We collected clinical data, including disease severity scores and quality of life indices, along with laboratory markers such as complete blood count and C-reactive protein. Smoking status and cumulative exposure (pack-years) were assessed based on patient history. To compare laboratory parameters between smoking subgroups, we used Mann–Whitney U tests. Additionally, we performed logistic regression analyses to evaluate the association between cumulative cigarette exposure and the presence of comorbidities. **Results**: Among the cohort, 63.4% were active smokers with a median of 15 pack-years. Smokers had significantly higher leukocyte, neutrophil, lymphocyte, monocyte, eosinophil, and basophil counts, indicating elevated systemic inflammation. Hematocrit, hemoglobin, mean corpuscular volume, and mean corpuscular hemoglobin were also significantly higher in smokers, while C-reactive protein levels did not differ notably between groups. Subgroup analysis revealed that patients with arterial hypertension, diabetes mellitus, and hypercholesterolemia had significantly more pack-years than those without these conditions. These comorbidities, components of metabolic syndrome, were strongly associated with higher lifetime tobacco exposure in HS patients. **Conclusions**: Smoking contributes not only to heightened inflammatory activity in HS but is also significantly associated with the presence of metabolic comorbidities. These findings underscore the importance of early interdisciplinary intervention and structured smoking cessation programs to improve outcomes in HS patients.

## 1. Introduction

Hidradenitis suppurativa (HS) is a chronic inflammatory skin condition characterized by recurrent painful nodules, abscesses, and fistulas [1]. The exact pathomechanism remains unclear, but genetic predisposition, environmental factors and a dysregulated immune response play a central role [2]. Follicle occlusion leads to rupture of the hair follicle and triggers a strong inflammatory response [3]. This is characterized by a dysregulation of the immune response, in particular by cytokines such as TNF-α, IL-1β and Th-17, which maintain the inflammatory processes [4]. The chronic course of the disease results in scarring, tissue destruction and significant impairment of quality of life [5].

HS is often associated with comorbidities such as obesity, which worsens the disease not only through mechanical factors but also through hormonal and inflammatory processes [6,7]. In addition, metabolic syndrome, consisting of hyperlipidemia, arterial hypertension (AHT) and insulin resistance, is common in affected individuals [6,8]. Chronic inflammation exacerbates these metabolic disturbances and increases the risk of cardiovascular disease [6]. Diabetes mellitus, caused by insulin resistance, is another common comorbidity [9,10]. Mental health issues such as depression and anxiety disorders are more common in patients with HS [11].

Smoking is a significant risk factor and worsens the course of HS [12,13,14,15]. Smokers show more severe disease progression and a poorer response to therapy [7,13,16]. The toxic substances in tobacco smoke promote oxidative stress and the release of pro-inflammatory cytokines such as TNF-α and IL-6, which further intensifies the chronic inflammation [17,18,19]. Laboratory analysis in smokers shows typical inflammatory changes compared to non-smokers. Elevated leukocyte counts, especially of neutrophils, indicate chronic inflammatory activity. Monocytes, which are involved in chronic inflammation, are also elevated, reflecting the systemic inflammation [20]. Increased erythrocyte count, hematocrit and hemoglobin levels are observed in smokers in response to carbon monoxide-induced hypoxia [21]. In addition, smokers have elevated C-reactive protein (CRP) levels, illustrating the increased systemic inflammatory response and highlighting the negative impact of smoking on disease progression [22,23]. Patients with a Hurley stage III disease often show a particularly strong inflammatory activity with strongly increased CRP values, so that the CRP value is used as a marker for the inflammatory activity in HS patients [24,25].

In addition, smoking delays wound healing due to increased neutrophil extracellular trap formation, which is problematic in a disease with abscesses and fistulas [26].

To date, the extent to which comorbidities are associated with lifetime pack-years in patients with HS has not been investigated. The aim of this study was to find out whether the number of cigarettes smoked differed between HS-patients with a specific comorbidity and those without. In addition, relevant differences in blood cell counts and CRP levels between smoking and non-smoking patients with HS were to be investigated.

## 2. Materials and Methods

### 2.1. Design and Setting

A retrospective, single-center study was carried out to assess the influence of smoking on HS. The study adhered to the ethical guidelines outlined in the Declaration of Helsinki. Approval for this research was obtained from the institutional ethics committee of the Ruhr-University Bochum (ethics approval: #5302/15).

### 2.2. Patients and Data Collection

We analyzed patient data regarding personal, disease-specific, and laboratory information. The laboratory parameters assessed encompassed a complete blood count and CRP. The severity of HS disease was evaluated using the Severity Assessment of Hidradenitis Suppurativa score (SAHS), Modified Hidradenitis Suppurativa Score (mHSS), and Hurley system. To assess patients’ quality of life, the Dermatology Life Quality Index (DLQI) questionnaire was administered. All comorbidities were recorded, with the ten most common ones listed in this paper. Smoking status and the number of tobacco pack-years were also determined. One pack-year is defined by smoking one pack of cigarettes per day for a period of one year [27]. The formula to calculate pack-years is:

Tobacco pack-years = (Number of cigarettes smoked per day ÷ 20) × Number of years smoked

Smoking status was classified as active-smoker, never-smoker and ex-smoker. As part of the investigation into whether there are significant differences in lab values between active smokers and never-smokers, ex-smokers were excluded due to a low sample size.

### 2.3. Data Analysis

We conducted statistical analyses using IBM SPSS Statistics (version 29.0) and R statistical software (version 4.2.1; R Core Team, 2023). A significance threshold of *p* < 0.05 was applied. Descriptive statistics were employed to outline the demographic and clinical profiles of the patients. The findings are expressed as counts (percentages), medians with ranges, or medians with interquartile ranges (IQR). The normality of the variables was evaluated via the Shapiro–Wilk test and by generating Q-Q plots. Mann–Whitney U tests were performed to identify differences between the subgroups. The graph was created using the GraphPad Prism software. To further explore these associations, multiple logistic regression analyses were conducted to examine predictors of obesity, diabetes mellitus, and AHT.

## 3. Results

### 3.1. Patient Characteristics

In this cohort of 131 patients with HS (Table 1), we observed a higher prevalence of the condition in females (58%) compared to males (42%). The median age of patients was 40 years, with an IQR of 32 to 51 years, while the median age of disease onset was 23 years (IQR: 18–31.5). The median duration of HS in these patients was 12 years (IQR: 6.5–23), reflecting the chronicity of the disease. The DLQI yielded a median score of 14, with a range from 0 to 30, indicating an impairment in quality of life among the patients. Notably, a significant proportion of patients were affected by obesity, with a median body mass index (BMI) of 31.35 kg/m^2^ (IQR: 27.2–36.5).

Disease severity was categorized using the Hurley staging system, where 48.1% of patients were classified as Hurley stage II (*n* = 63), and 46.6% as stage III (*n* = 61), indicative of advanced disease. The median mHSS was 40 (IQR: 19.5–75.5), further highlighting the significant burden of the disease on this patient population.

Smoking status emerged as a key factor, with 63.4% of patients being active smokers, reporting a median of 15 pack-years (IQR: 9.3–30). A smaller portion (6.1%) were ex-smokers, while 30.5% of the cohort identified as non-smokers. Additionally, a positive family history of HS was reported by 32.1% of patients.

### 3.2. Differences of Laboratory Parameters Between Smokers and Never-Smokers with HS

A comparative analysis of laboratory parameters between never-smoking and smoking patients with HS revealed significant variations, particularly in markers of inflammation (Table 2). Smokers exhibited markedly higher leukocyte counts (median 10,100 cells/µL, IQR: 8450–12,377.5) compared to never-smokers (median 7120 cells/µL, IQR: 6110–9910), with a statistically significant difference (*p* < 0.001). This trend extended to neutrophils (median 6415 cells/µL in smokers vs. 4650 cells/µL in never-smokers, *p* < 0.001), lymphocytes (median 2660 cells/µL in smokers vs. 1760 cells/µL in never-smokers, *p* < 0.001), and monocytes (median 665 cells/µL in smokers vs. 510 cells/µL in never-smokers, *p* < 0.001). Eosinophils and basophils were also significantly elevated in smokers (*p* = 0.017 and *p* = 0.003, respectively).

Additionally, smokers had higher hematocrit levels (median 42.1%, IQR: 39.8–44.78) compared to never-smokers (median 40.7%, IQR: 37.4–42.7, *p* = 0.017), and hemoglobin levels were similarly increased in smokers (median 14 g/dL, IQR: 13.45–15.08) compared to never-smokers (median 13.2 g/dL, IQR: 12.7–14.5, *p* = 0.004). Mean corpuscular volume (MCV) and mean corpuscular hemoglobin (MCH) were also significantly higher in smokers (*p* = 0.039 and *p* = 0.023, respectively). However, no significant differences were observed in erythrocyte count, mean corpuscular hemoglobin concentration (MCHC), platelet count, or CRP levels between the two groups.

### 3.3. Differences in Pack-Years in the Presence of Various Comorbidities

**Figure 1:**
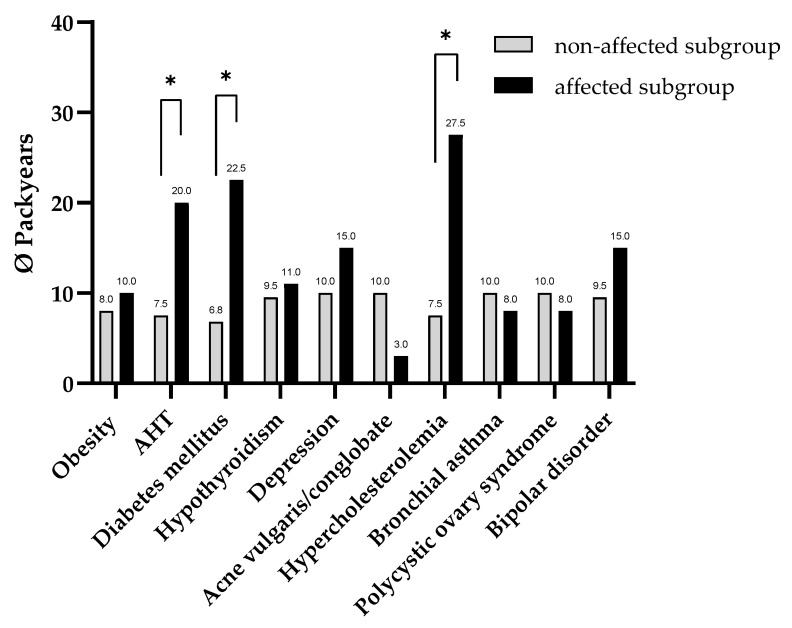
Comparison of cumulative cigarette exposure (pack-years) between HS patients with and without comorbidities (*n* = 131). Patients with comorbidities show significantly higher median pack-years than those without (*p* < 0.05), indicating a potential association between smoking intensity and comorbidity development in HS. Asterisk (*) indicates statistical significance at α = 0.05.

A subgroup analysis of HS patients explored the association between smoking history, measured in pack-years, and the presence of various comorbidities. Significant differences were observed in patients with AHT, diabetes mellitus, and hypercholesterolemia, all of which demonstrated a markedly higher smoking burden (Figure 1). Patients with AHT exhibited a median of 20 pack-years (IQR: 5–33), compared to 7.5 pack-years (IQR: 0–15) in those without AHT (*p* = 0.005). Similarly, diabetic patients had a median of 22.5 pack-years (IQR: 9.75–46.25), significantly higher than the 7 pack-years (IQR: 0–20) seen in non-diabetic patients (*p* = 0.001). Hypercholesterolemia was also associated with increased smoking exposure, with affected patients showing a median of 27.5 pack-years (IQR: 16.25–47.13), in contrast to 7.5 pack-years (IQR: 0–20) in patients without the condition (*p* = 0.003). To further explore these associations, multiple logistic regression analyses were conducted to examine predictors of obesity, diabetes mellitus, and AHT (Appendix A).

#### 3.3.1. Diabetes Mellitus

Obesity was a robust predictor of diabetes across both models (Model 1: Odds Ratio (OR) = 5.63, 95% CI [1.56, 27.48], *p* = 0.015; Model 2: OR = 7.39, 95% CI [1.90, 42.53], *p* = 0.009). In addition, age (OR = 1.09, 95% CI [1.03, 1.15], *p* = 0.002) and Hurley stage III (Model 2: OR = 3.93, 95% CI [1.20, 14.34], *p* = 0.028) were significantly associated with higher odds of diabetes. Pack-years also predicted diabetes in Model 2 (OR = 1.04, 95% CI [1.02, 1.07], *p* = 0.003). Adalimumab, sex, and AHT were not significant predictors in either model (Appendix A).

#### 3.3.2. Arterial Hypertension (AHT)

In both models, obesity significantly increased the likelihood of AHT (Model 1: OR = 3.58, 95% CI [1.34, 10.62], *p* = 0.015; Model 2: OR = 3.47, 95% CI [1.34, 9.96], *p* = 0.014). Age was also a consistent predictor (Model 1: OR = 1.08, 95% CI [1.04, 1.13], *p* = 0.001), and pack-years was significant in Model 2 (OR = 1.02, 95% CI [1.00, 1.05], *p* = 0.038). Hurley stage III reached significance only in Model 2 (OR = 2.62, 95% CI [1.06, 6.73], *p* = 0.040). No significant associations were found for sex, diabetes mellitus, or Adalimumab in predicting AHT (Appendix A).

#### 3.3.3. Obesity

Two models were estimated. In both, diabetes mellitus (Model 1: OR = 5.98, 95% CI [1.69, 28.57], *p* = 0.011; Model 2: OR = 6.90, 95% CI [1.88, 35.81], *p* = 0.008) and AHT (Model 1: OR = 3.43, 95% CI [1.30, 10.03], *p* = 0.017; Model 2: OR = 3.35, 95% CI [1.30, 9.54], *p* = 0.016) emerged as significant predictors of obesity. Other variables, including age, sex, Hurley stage III, and Adalimumab treatment, were not statistically significant in either model. Pack-years did not reach statistical significance in Model 2 (*p* = 0.123) (Appendix A).

## 4. Discussion

Our data analysis indicates that HS patients with AHT, hypercholesterolemia, or diabetes mellitus have a higher number of pack-years compared to HS patients without these comorbidities. This finding indicates that the quantity of pack-years accumulated over the course of an individual’s life represents a substantial risk factor for the emergence of these comorbidities in HS patients.

Our comparative analysis of smoking HS patients and non-smoking HS patients reveals a statistically significant increase in the overall leukocyte count, encompassing various subtypes such as neutrophil, lymphocyte, monocyte, eosinophil, and basophil leukocytes, among the former group. This increase in leukocyte counts can be attributed to several biochemical mechanisms. These include the activation of the NF-κB signaling pathway [28], the increased release of proinflammatory cytokines [29], the activation of macrophages [30], nicotine-induced adrenaline release in the adrenal medulla [31] and the erythropoietin-mediated increase in hematopoiesis [32]. In essence, these processes are manifestations of a pro-inflammatory stimulus instigated by tobacco consumption. Chronic inflammatory state have deleterious effects on the course of HS, as well as contribute to the pathogenesis and progression of AHT, hypercholesterolemia, and diabetes mellitus [33,34].

Interestingly the CRP-level did not vary between smokers and non-smokers in this study. In this cohort with many Hurley II–III patients, the inflammatory baseline is very high, creating a ceiling effect that leaves little room for smoking to further increase CRP.

Patients diagnosed with AHT and hypercholesterolemia exhibit an elevated risk of developing atherosclerotic changes and endothelial dysfunction due to the augmented vascular load [35]. In patients diagnosed with HS, the presence of chronic systemic inflammation has been demonstrated to elevate the risk of cardiovascular incidents, including myocardial infarction and stroke, as well as augmented cardiovascular mortality [36,37]. It has been demonstrated that tobacco consumption exerts a vasculotoxic effect, leading to the promotion of vasoconstriction, elevated blood pressure, and deleterious effects on lipid profiles [38]. The collective impact of these risk factors may elucidate the heightened severity of disease manifestations observed in HS patients who smoke. The deleterious effects of tobacco-related AHT and hypercholesterolemia on vascular integrity may compound the adverse impact on local microcirculation within tobacco-affected skin regions, thereby impeding wound healing in these areas [39,40].

In addition to impairing local microcirculation and wound healing, tobacco use may contribute directly to fibrosis in HS-affected skin through mechanisms analogous to those observed in pulmonary and hepatic fibrogenesis [41]. Nicotine has been shown to modulate fibroblast behavior in multiple organ systems, promoting their survival while impairing their migration, thereby leading to fibroblast accumulation at the wound edge and excessive extracellular matrix deposition [42,43,44]. In the skin, this dysregulation of fibroblast function—characterized by reduced collagen turnover and increased matrix metalloproteinase-8 activity—contributes to aberrant wound healing and may drive excessive scarring and fistula tract formation, which are hallmarks of advanced HS [45]. Furthermore, keratinocyte migration, essential for re-epithelialization, is disrupted through nicotine’s effect on cholinergic signaling, particularly via α7 nicotinic acetylcholine receptors, which inhibit cell motility by modulating integrin expression and Rho-kinase activity [46,47]. Taken together, these findings suggest that smoking not only fuels chronic inflammation but may also actively promote fibrotic remodeling of HS lesions, exacerbating disease severity and the formation of chronic sinus tracts.

Diabetes mellitus, AHT and hypercholesterolemia are part of the metabolic syndrome, the etiology of which is influenced by a number of factors in the lifestyle [48]. These include, in particular, physical inactivity, an unhealthy diet and tobacco consumption. In consideration of physical inactivity, it has been observed that patients afflicted with severe metabolic diseases frequently adopt a more sedentary lifestyle as a consequence of their illness [49]. The potential for adverse lifestyle habits to contribute significantly to the co-occurrence of these comorbidities and smoking behavior is a plausible hypothesis that merits further investigation. It has been posited that a high number of pack-years could serve as an indicator of an unhealthy lifestyle.

Additionally, chronic diseases such as HS have been associated with significant psychological distress and persistent feelings of stress [50,51]. Prolonged exposure to stress has been demonstrated to result in elevated serum cortisol levels. This, in turn, has been shown to engender a decline in insulin sensitivity over time, thereby promoting visceral obesity and elevating blood pressure [52,53]. It has been documented that certain patients utilize tobacco use as a subjective coping strategy to reduce stress [54]. Accordingly, 63.4% of all patients in our study reported currently smoking cigarettes. In comparison, the estimated prevalence of smoking in the general population in Germany is approximately 30% [55]. This suggests that patients with HS in Germany may smoke excessively. However, tobacco use has been demonstrated to contribute to an increase in inflammatory markers and oxidative stress over time, which can further exacerbate physiological stress [56]. The combination of chronic stress and increased tobacco use has been shown to result in a further increase in the severity of HS and the risk of associated comorbidities. This combination represents a reinforcing element in the pathophysiological vicious circle.

In patients with a BMI > 35, a correlation was identified between the duration of cigarette smoking and the severity of HS. Conversely, the onset of the disease is frequently observed at a significantly younger age in individuals with a positive family history [57]. This clinical heterogeneity suggests that subtyping HS could be useful. One possible subtype is the ‘metabolic type’, which is characterized by pronounced metabolic inflammation and an elevated BMI. Patients with HS exhibit increased insulin resistance, which is most likely mediated by epigenetic modifications [10]. Smoking is also associated with an increased risk of insulin resistance [58]. Cumulative effects are a plausible explanation for our findings and may have deleterious consequences, particularly in individuals with the ‘metabolic phenotype’.

Beyond heightened meta-inflammation due to epigenetic factors in HS, genetics may further contribute to the increased risk of selected comorbidities. Accordingly, a comprehensive analysis of 391,481 individuals residing in the United Kingdom has indicated that genetic factors play a substantial role in determining the risk of developing coronary heart disease, diabetes mellitus, and alterations in the plasma proteome in specific HS patients [59].

In patients with HS who also have metabolic comorbidities, the targeted treatment of these concomitant diseases could also contribute to a reduction in the severity of HS. For instance, the utilization of the antidiabetic pharmaceutical metformin in patients diagnosed with type 2 diabetes mellitus and concomitant HS has demonstrated a clinical improvement in cutaneous manifestations [60,61]. Concurrently, it is imperative to acknowledge that a significant proportion of patients afflicted with comorbidities, including but not limited to diabetes mellitus and AHT, receive prolonged medication regimens. These medications have the potential to exert a deleterious effect on the progression of HS. For instance, statins have been associated with an elevated risk of soft tissue infections [62].

The findings of this study indicate that the presence of metabolic syndrome should always be considered in smokers with HS. In order to circumvent a prognostically unfavorable course, it appears necessary to implement early interdisciplinary care, which should include structured offers for tobacco cessation. However, studies demonstrate that patients diagnosed with HS exhibit no notable increase in the propensity to discontinue smoking when compared to smokers without HS [63]. Chronic diseases, including HS and diabetes mellitus, have been demonstrated to be associated with significant psychological distress, which can adversely affect an individual’s capacity to sustain smoking cessation efforts. In this context, smoking could be considered a short-term coping strategy for emotional stress, particularly in patients with co-existing metabolic comorbidities. This phenomenon may perpetuate a detrimental cycle, wherein smoking is employed as a stress-relieving mechanism, leading to an escalation in inflammatory disease symptoms and an accumulation of nicotine exposure.

To reinforce patients’ decision to quit smoking the ARRIBA score has been recognized as a reliable instrument for visualizing individual cardiovascular risk in cardiology [64]. The development of a risk assessment model analogous to the ARRIBA score for HS is a conceivable prospect. One potential application of such a model which may include factors such as laboratory parameter and coexistence of comorbidities would be to elucidate the impact of risk factors, such as smoking, on the progression of the disease. The integration of a graphical representation has been demonstrated to enhance patients’ risk perception and augment their motivation to cease smoking [65].

A prospective study demonstrated that the leukocyte count exhibits a significant decrease following the cessation of smoking, reaching a level that is marginally above the mean value observed in individuals who have never smoked [66]. Although ex-smokers were excluded from the statistical analysis of laboratory parameters in our study due to insufficient patient numbers, a positive effect of smoking cessation can be assumed. Therefore, we strongly recommend that all patients with HS who smoke receive structured smoking-cessation support, which may help limit disease worsening and reduce the burden of associated metabolic comorbidities.

Some methodological and contextual limitations should be taken into account when interpreting the findings of this study. First, its retrospective and observational design inherently precludes causal inference between smoking and the presence of metabolic comorbidities. The cross-sectional nature of the data further limits the ability to assess temporal relationships or the progression of these comorbidities in relation to cumulative tobacco exposure.

Smoking status and pack-year estimates were based on self-report, introducing the potential for recall bias and underreporting, particularly due to social desirability. Furthermore, important lifestyle factors such as dietary habits, alcohol consumption, and physical activity were not assessed and may represent unmeasured confounders influencing the observed associations.

The study was conducted at a single tertiary care center with a patient population skewed toward more severe disease phenotypes. This monocentric and selective setting may introduce center-related bias and limit the generalizability of the findings to broader or less severely affected HS populations.

Additionally, the overall sample size was relatively small, which may reduce statistical power and limit the robustness of subgroup analyses. In particular, the low number of former smokers in the cohort precluded meaningful stratification or analysis of this subgroup, thereby limiting the applicability of findings across the full spectrum of smoking behavior.

## 5. Conclusions

In summary, our observations indicate that smoking should not be regarded as a solitary risk factor for HS; rather, it should be considered as part of a multifaceted relationship with comorbidities such as diabetes mellitus, AHT, and hypercholesterolemia. Further research is necessary to investigate whether the higher number of pack-years in these patient groups is actually causally linked to the comorbidities or whether common risk factors such as an unhealthy lifestyle or socioeconomic factors are underlying causes. In any event, smoking abstinence should be the objective, and support for the patient should be considered if necessary.

## Figures and Tables

**Table 1:** Personal characteristics and disease-specific characteristics of patients with HS.

Parameters	Values
Female sex, *n* (% ^b^)	55 (58)
Male sex, *n* (% ^b^)	76 (42)
Age, median (IQR), y	40 (32–51)
Age of HS onset, median (IQR), y	23 (18–31.5)
Duration of HS, median (IQR), y	12 (6.5–23)
BMI, median (IQR), kg/m^2^	31.35 (27.2–36.5)
Positive family history of HS, *n* (% ^b^)	42 (32.1)
Negative family history of HS, *n* (% ^b^)	89 (67.9)
Never-smoker, *n* (% ^b^)	40 (30.5)
Never-smoker pack-years, median (IQR)	0 (0–0)
Active smoker, *n* (% ^b^)	83 (63.4)
Active-smoker pack-years, median (IQR)	15 (9.3–30)
Ex-smoker, *n* (% ^b^)	8 (6.1)
Ex-smoker pack-years, median (IQR)	20 (9.8–20)
Hurley I, *n* (% ^b^)	7 (5.3)
Hurley II, *n* (% ^b^)	63 (48.1)
Hurley III, *n* (% ^b^)	61 (46.6)
SAHS, median (IQR)	8 (5–9)
mSS, median (IQR)	40 (19.5–75.5)
DLQI, median (range)	14 (0–30)
Current pain on a visual analogue scale, median (IQR)	3 (0–10)
Flare-ups in the last 4 weeks, median (range)	0 (0–12)
Involvement of axilla, *n* (% ^b^)	91 (67.5)
Involvement of mammary, *n* (% ^b^)	24 (18.3)
Involvement of abdomen, *n* (% ^b^)	14 (10.7)
Involvement of mons pubis, *n* (% ^b^)	46 (35.1)
Involvement of inguinal, *n* (% ^b^)	75 (57.3)
Involvement of genital, *n* (% ^b^)	40 (30.5)
Involvement of perianal, *n* (% ^b^)	21 (16.1)
Involvement of gluteal, *n* (% ^b^)	37 (28.2)
Upper thigh, *n* (% ^b^)	29 (22.1)
Obesity ^a^, *n* (% ^b^)	79 (60.3)
AHT ^a^, *n* (% ^b^)	37 (28.2)
Diabetes mellitus ^a^, *n* (% ^b^)	24 (18.3)
Hypothyroidism ^a^, *n* (% ^b^)	19 (14.5)
Depression ^a^, *n* (% ^b^)	16 (12.2)
Acne vulgaris/conglobata ^a^, *n* (% ^b^)	15 (11.5)
Hypercholesterolemia ^a^, *n* (% ^b^)	14 (10.7)
Bronchial asthma ^a^, *n* (% ^b^)	8 (6.1)
Polycystic ovary syndrome ^a^, *n* (% ^b^)	4 (3.1)
Bipolar disorder ^a^, *n* (% ^b^)	4 (3.1)

Abbreviations: *n* = absolute number of patients; IQR = interquartile range; y = years; HS = Hidradenitis suppurativa; BMI = body mass index; SAHS = Hidradenitis Suppurativa score; mHSS = modified Hidradenitis Suppurativa Score; DLQI = Dermatology Life Quality Index; AHT = Arterial hypertension. ^a^ Ten most common comorbidities in our cohort ^b^ percentages use: *n* = 131 unless otherwise specified.

**Table 2:** Analysis of laboratory parameters in non-smoking and smoking patients with HS a (continuous variables with Mann–Whitney U-Test).

Laboratory Parameters	Never-Smokers ^a^	Active Smokers ^b^	*p*-Value
Leukocytes, median (IQR), Cells/µL	7120 (6110–9910)	10,100 (8450–12,377.5)	<0.001 *
Neutrophils, median (IQR), Cells/µL	4650 (3830–6460)	6415 (4805–8377.5)	<0.001 *
Lymphocytes, median (IQR), Cells/µL	1760 (1360–2150)	2660 (2157.5–3237.5)	<0.001 *
Monocytes, median (IQR), Cells/µL	510 (410–720)	665 (572.5–837.5)	<0.001 *
Eosinophils, median (IQR), Cells/µL	110 (60–170)	170 (92.5–267.5)	0.017 *
Basophils, median (IQR), Cells/µL	40 (30–50)	50 (40–70)	0.003 *
Erythrocytes, median (IQR), Cells/µL	4.65 (4.3–5.24)	4.7 (4.4–5.08)	0.56
Hematocrit, median (IQR), %	40.7 (37.4–42.7)	42.1 (39.8–44.78)	0.017 *
Hemoglobin, median (IQR), g/dL	13.2 (12.7–14.5)	14 (13.45–15.08)	0.004 *
MCV, median (IQR), fl	87.1 (82.6–89.5)	88.3 (85.2–91.9)	0.039 *
MCH, median (IQR), pg	28.9 (27.1–30.4)	29.95 (28.5–31)	0.023 *
MCHC, median (IQR), g/dL	33.2 (32.5–34.2)	33.7 (32.8–34.4)	0.327
Platelets, median (IQR), Cells/µL	306,000 (264,000–341,000)	293,000 (249,250–344,750)	0.94
CRP, median (IQR), mg/L	5 (5–16.5)	6 (5–14.1)	0.2

Abbreviations: IQR = interquartile range; MCV = mean corpuscular volume; MCH = mean corpuscular hemoglobin; MCHC = mean corpuscular hemoglobin concentration; CRP = C-reactive protein; ^a^ number of patients who never smoke: 40; ^b^ number of patients who do smoke: 83; *p* < 0.05 is marked with an asterisk (*) and highlighted in bold.

## Data Availability

Data from study are available on request from the corresponding author due to privacy.

## Abbreviations

The following abbreviations are used in this manuscript:

AHT	Arterial Hypertension
BMI	Body Mass Index
CI	Confidence Interval
CRP	C-reactive Protein
DLQI	Dermatology Life Quality Index
HS	Hidradenitis Suppurativa
IQR	Interquartile Range
MCH	Mean Corpuscular Hemoglobin
MCHC	Mean Corpuscular Hemoglobin Concentration
MCV	Mean Corpuscular Volume
mHSS	Modified Hidradenitis Suppurativa Score
NF-κB	Nuclear Factor kappa-light-chain-enhancer of activated B cells
IL-1β	Interleukin 1 beta
IL-6	Interleukin 6
OR	Odds Ratio
SAHS	Severity Assessment of Hidradenitis Suppurativa
Th-17	T helper 17 cells
TNF-α	Tumor Necrosis Factor alpha

## Appendix A

**Table A1:** Subgroup analysis of pack-years in HS patients with and without different comorbidities (Continuous variables with Mann–Whitney U-Test).

Comorbidities ^a^, *n* (% ^c^)	Non-Affected		Affected		*p*-Value
	Amount ^b^ [% ^c^]	Pack-Years, Median [IQR]	Amount ^b^ [% ^c^]	Pack-Years, Median [IQR]	
Obesity	52 (39.7)	8 (0–20)	79 (60.3)	10 (0–20)	0.746
AHT	94 (71.8)	7.5 (0–15)	37 (28.3)	20 (5–33)	**0.005 ***
Diabetes mellitus	107 (81.7)	7 (0–20)	24 (18.3)	22.5 (9.75–46.25)	**0.001 ***
Hypothyroidism	112 (85.5)	9.5 (0–20)	19 (14.5)	11 (2.5–20)	0.722
Depression	115 (87.8)	10 (0–20)	16 (12.2)	15 (5.15–30)	0.151
Acne vulgaris/conglobate	116 (88.5)	10 (0–20)	15 (11.5)	3 (0–11.5)	0.117
Hypercholesterolemia	117 (89.3)	7.5 (0–20)	14 (10.7)	27.5 (16.25–47.13)	**0.003 ***
Bronchial asthma	122 (93.9)	10 (0–20)	8 (6.1)	8 (3.94–11.25)	0.625
Polycystic ovary syndrome	127 (96.9)	10 (0–20)	4 (3.1)	8 (2.25–13.5)	0.607
Bipolar disorder	127 (96.9)	10 (0–20)	4 (3.1)	25 (10–45)	0.121

Abbreviations: *n* = absolute number of patients; AHT = arterial hypertension; IQR = interquartile range. *p* value: * significant result (*p* < 0.05) ^a^ Ten most common comorbidities in our cohort ^b^ Total number of HS patients: 131 ^c^ percentages use: *n* = 131 unless otherwise specified. *p* < 0.05 is marked with an asterisk (*) and highlighted in bold.

**Table A2:** Results of the multiple logistic regression predicting diabetes mellitus in model 1.

Predictor	B	SE	z	*p*	OR	95% CI OR
(Intercept)	−7.21	1.55	−4.67	<0.001	0.00	[0.00, 0.01]
Age	**0.08 ***	0.03	3.11	**0.002 ***	1.09	[1.03, 1.15]
Sex (female)	−0.71	0.60	−1.19	0.236	0.49	[0.14, 1.55]
Hurley Stage III	1.23	0.61	2.03	0.043	3.42	[1.08, 12.07]
Adalimumab (active)	0.50	0.70	0.71	0.475	1.64	[0.40, 6.35]
Obesity	**1.73 ***	0.71	2.43	**0.015 ***	5.63	[1.56, 27.48]
AHT	0.35	0.59	0.60	0.552	1.42	[0.44, 4.42]

Legend: AHT = arterial hypertension; B = regression coefficient (log odds); SE = standard error; z = z-value (Wald statistic); *p* = *p*-value (APA style: no leading zero, e.g., 0.011); OR = odds ratio (exp(B)); CI = 95% confidence interval of the odds ratio, *p* < 0.05 is marked with an asterisk (*) and highlighted in bold.

**Table A3:** Results of the multiple logistic regression predicting diabetes mellitus in model 2.

Predictor	B	SE	z	*p*	OR	95% CI OR
(Intercept)	−4.68	0.99	−4.72	<0.001	0.01	[0.00, 0.05]
Pack-years	**0.04 ***	0.01	2.96	**0.003 ***	1.04	[1.02, 1.07]
Sex (female)	−0.44	0.60	−0.74	0.461	0.64	[0.19, 2.05]
Hurley Stage III	**1.37 ***	0.62	2.20	**0.028 ***	3.93	[1.20, 14.34]
Adalimumab (active)	0.40	0.68	0.59	0.554	1.50	[0.37, 5.63]
Obesity	**2.00 ***	0.77	2.60	**0.009 ***	7.39	[1.90, 42.53]
AHT	0.59	0.57	1.04	0.300	1.81	[0.58, 5.53]

Legend: AHT = arterial hypertension; B = regression coefficient (log odds); SE = standard error; z = z-value (Wald statistic); *p* = *p*-value (APA style: no leading zero, e.g., 0.011); OR = odds ratio (exp(B)); CI = 95% confidence interval of the odds ratio, *p* < 0.05 is marked with an asterisk (*) and highlighted in bold.

**Table A4:** Results of the multiple logistic regression predicting arterial hypertension in model 1.

Predictor	B	SE	z	*p*	OR	95% CI OR
(Intercept)	−5.71	1.18	−4.84	<0.001	0.00	[0.00, 0.03]
Age	**0.08 ***	0.02	3.40	**0.001 ***	1.08	[1.04, 1.13]
Sex (female)	0.51	0.47	1.08	0.282	1.67	[0.66, 4.32]
Hurley Stage III	0.79	0.48	1.62	0.105	2.19	[0.85, 5.79]
Adalimumab (active)	−0.51	0.64	−0.79	0.429	0.60	[0.16, 2.02]
Obesity	**1.28 ***	0.52	2.44	**0.015 ***	3.58	[1.34, 10.62]
Diabetes mellitus	0.33	0.57	0.58	0.564	1.39	[0.45, 4.26]

Legend: B = regression coefficient (log odds); SE = standard error; z = z-value (Wald statistic); *p* = *p*-value (APA style: no leading zero, e.g., 0.011); OR = odds ratio (exp(B)); CI = 95% confidence interval of the odds ratio, *p* < 0.05 is marked with an asterisk (*) and highlighted in bold.

**Table A5:** Results of the multiple logistic regression predicting arterial hypertension in model 2.

Predictor	B	SE	z	*p*	OR	95% CI OR
(Intercept)	−2.98	0.63	−4.72	<0.001	0.05	[0.01, 0.16]
Pack-years	**0.02 ***	0.01	2.08	**0.038 ***	1.02	[1.00, 1.05]
Sex (female)	0.64	0.46	1.40	0.162	1.90	[0.78, 4.79]
Hurley Stage III	**0.96 ***	0.47	2.06	**0.040 ***	2.62	[1.06, 6.73]
Adalimumab (active)	−0.57	0.61	-0.93	0.353	0.57	[0.16, 1.79]
Obesity	**1.24 ***	0.51	2.46	**0.014 ***	3.47	[1.34, 9.96]
Diabetes mellitus	0.62	0.55	1.12	0.263	1.86	[0.62, 5.56]

Legend: B = regression coefficient (log odds); SE = standard error; z = z-value (Wald statistic); *p* = *p*-value (APA style: no leading zero, e.g., 0.011); OR = odds ratio (exp(B)); CI = 95% confidence interval of the odds ratio, *p* < 0.05 is marked with an asterisk (*) and highlighted in bold.

**Table A6:** Results of the multiple logistic regression predicting obesity in model 1.

Predictor	B	SE	z	*p*	OR	95% CI OR
(Intercept)	0.92	0.75	1.24	0.217	2.52	[0.579, 11.24]
Age	−0.02	0.02	−0.99	0.320	0.98	[0.95, 1.02]
Sex (female)	−0.33	0.40	−0.81	0.417	0.72	[0.33, 1.59]
Hurley Stage III	−0.57	0.43	−1.34	0.181	0.56	[0.24, 1.29]
Adalimumab (active)	0.60	0.61	0.99	0.323	1.83	[0.57, 6.44]
Diabetes mellitus	1.79 *	0.70	2.55	0.011 *	5.98	[1.69, 28.57]
AHT	**1.23 ***	0.52	2.39	**0.017 ***	3.43	[1.30, 10.03]

Legend: AHT = arterial hypertension; B = regression coefficient (log odds); SE = standard error; z = z-value (Wald statistic); *p* = *p*-value (APA style: no leading zero, e.g., 0.011); OR = odds ratio (exp(B)); CI = 95% confidence interval of the odds ratio, *p* < 0.05 is marked with an asterisk (*) and highlighted in bold.

**Table A7:** Results of the multiple logistic regression predicting obesity in model 2.

Predictor	B	SE	z	*p*	OR	95% CI OR
(Intercept)	0.45	0.36	1.26	0.209	1.56	[0.79, 3.19]
Pack-years	−0.02	0.01	−1.54	0.123	0.98	[0.96, 1.00]
Sex (female)	−0.37	0.41	−0.92	0.358	0.69	[0.31, 1.52]
Hurley Stage III	−0.58	0.43	−1.37	0.172	0.56	[0.24, 1.27]
Adalimumab (active)	0.58	0.61	0.95	0.340	1.79	[0.56, 6.30]
Diabetes mellitus	**1.93 ***	0.73	2.64	**0.008 ***	6.90	[1.88, 35.81]
AHT	**1.21 ***	0.50	2.41	**0.016 ***	3.35	[1.30, 9.54]

Legend: AHT = arterial hypertension; B = regression coefficient (log odds); SE = standard error; z = z-value (Wald statistic); *p* = *p*-value (APA style: no leading zero, e.g., 0.011); OR = odds ratio (exp(B)); CI = 95% confidence interval of the odds ratio, *p* < 0.05 is marked with an asterisk (*) and highlighted in bold.
